# Flow Immersive: A Multiuser, Multidimensional, Multiplatform Interactive Covid-19 Data Visualization Tool

**DOI:** 10.3389/fpsyg.2021.661613

**Published:** 2021-05-13

**Authors:** Michael DiBenigno, Mehmet Kosa, Mina C. Johnson-Glenberg

**Affiliations:** ^1^Flow Immersive, Mountain View, CA, United States; ^2^Department of Psychology, Arizona State University, Tempe, AZ, United States

**Keywords:** data simulation, data visualization, Covid-19, augmented reality, virtual reality, spatial design

## Abstract

Covid-19 has prompted a surge of data visualizations that have been published for public consumption, yet, many have not had broad appeal or may have not been well-understood by laypeople. A data storytelling platform called Flow Immersive has been created to successfully engage both laypeople and experts in understanding complex information. This tool integrates emerging technologies [e.g., augmented reality (AR) and virtual reality (VR)] with a multiplatform, multiuser publishing approach. From October 2020 to December 2020, Flow’s Covid-19 AR videos captured 9 million (9,000,000) views, and have been used in multiple professional presentations. This paper documents the journey from development to deployment, and some user feedback which all led to breakthroughs in scalability and higher levels of engagement.

## Introduction

Design is the thoughtful organization of resources to accomplish a goal. It is both a process (i.e., set of activities) and a product (Hevner et al., 2004). During design and iteration, the search is about finding satisfactory solutions, i.e., satisficing without explicitly specifying all possible solutions (Simon, 1996). The design of data visualization has multiple solutions. We find it an intriguing and important space to work in, because data visualization literacy is a crucial skill for an educated populace (Börner et al., 2019). Visual and spatial presentation of health data is gaining prominence in general (Cicalò and Valentino, 2019), and it seems more timely than ever due to the Covid-19 pandemic because large segments of society that never thought much about virus transmission now want to understand how individuals and communities can best mitigate the pandemic’s toll. As people better understand data visualizations related to Covid-19, they may positively change their behaviors. In this paper, we describe a tool that visualizes up-to-date Covid-19 data and makes the three-dimensional (3D) information accessible for the public. We describe the multitude of ways Flow Immersive visualizations (called *Flows*) have been presented, and the impact of these various presentations.

While 3D is one feature, there are other design considerations made in the development of visualization tools. One consideration is utilizing scale to show the “zoomed out” big picture view, and then allowing users to then “zoom in” to the details. One can see the forest *and* the trees. Flow was designed so that data points do not disappear and reappear but instead move from one perspective, or graph, to another. Lastly, the use of steps allows participants to follow a linear storyline. At each step, participants can click on dots (data points), and pull-up additional details that aid in understanding a specific attribute. These feature and design decisions make the tool highly interactive and profoundly extensible.

As of this writing (February, 2021), the world is still inundated with daily, new content aimed at informing the general public about Covid-19-related topics. These visualizations can miss their mark by sometimes being confusing, or misunderstood by the layperson (see Romano et al., 2020 for comparison on linear and log scales). Many visualizations use traditional widgets such as two-dimensional (2D) bar and pie charts (which notoriously misrepresent area, see Cleveland, 1987). We have found several that are limited to certain regions, cities, and sometimes specific to only one state, city, or county (Arneson et al., 2020; Kaul et al., 2020; Mondal et al., 2020; Tebé et al., 2020; Zuo et al., 2020). The goal of the 3D data storytelling tool, Flow Immersive, was to create an extensible, generalizable Covid-19 visualization interface that would not be location specific and which would be accessible to multiple users through multiple devices or platforms.

## Adding a Third Dimension

**Figure 1:**
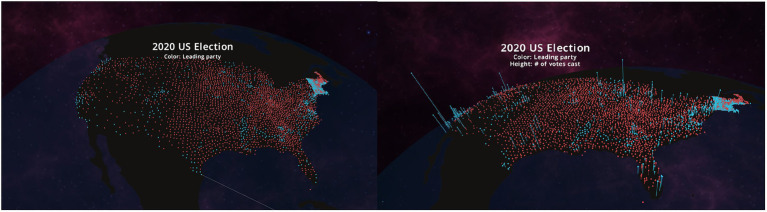
**Panel on the left** is two-dimensional (2D) and makes the United States appear to have mainly voted Republican (red) in 2020; **Panel on the right** with a third dimension of number of people is able to highlight the difference between rural and city voting patterns, as seen by the blue spikes over cities. This Flow story was shown very soon after the November, 2020 election to spur discussion around the difference in voting patterns between rural and urban areas.

Three-dimensional data visualization tools have been implemented for augmented reality (AR) and virtual reality (VR) in several domains such as civil engineering, industrial engineering, construction, and science in general (Bryson, 1996; Bruno et al., 2006; Schall et al., 2009; Donalek et al., 2014). Similarly, the engaging immersivity of AR and VR has increased learning in topics as varied as history (Blazauskas et al., 2017), medicine (Pennefather and Krebs, 2019), biology (Lee et al., 2010), and natural selection (Johnson-Glenberg et al., 2020). Several studies report that greater learning gains occur when content is learned in 3D VR compared to 2D desktop versions (Allcoat and von Mühlenen, 2018; Greenwald et al., 2018; Krokos et al., 2019; Johnson-Glenberg et al., 2020; Wu et al., 2020). Additionally, visualizing data in AR and VR can increase user collaboration (Meiguins et al., 2006) and aid in eliminating the fish tank problem (looking at the data behind a glass; Thomas et al., 2014). Providing immersive experiences consequently increases the understanding of the data (Bayyari and Tudoreanu, 2006). Adding a third dimension spatially reveals critical information that is otherwise lost with flat choropleths that use color bin ranges. For example, in a 2D map of America, showing votes cast by county (see Figure 1, left panel), it would appear that America is a predominantly red (Republican) country. However, land does not vote. When the third dimension, a height axis, is added, it reveals the number of votes in an area. It becomes evident that large cities are primarily blue (Figure 1, right panel). It is the actions of the inhabitants that drive the total countrywide count.

Immersion can be linked to the number of dimensions available to a viewer (Slater, 2003). We predict that the more immersed people are, the more motivated and engaged they will be (Makransky et al., 2019), and the better they should comprehend the Covid-19 data. Therefore, one other aim in our design was to make sure our tool was usable on the emerging and highly immersive technologies of AR and VR, both with mobile and head-mounted displays (HMD’s).

## The Tool: Flow Immersive

Flow Immersive, the data visualization design tool that was founded in 2016, enables users to more effectively communicate complex information and data stories to both expert and non-expert audiences.^1^ The tool enables users to tell data-driven narratives in an engaging, interactive, and understandable way, and is capable of creating a diverse set of data simulations (Flow Immersive, 2020).^2^

To date, Flow Immersive has created and published 17 interactive, 3D, Covid-19 data simulations that work across the following platforms: computer screens, mobile phones, VR headsets, AR mobile, and AR headsets. These simulations, called *Flows*, can be published as videos, images, GIFS, standalone visualizations, or immersive multiuser experiences. Additionally, these simulations work natively in a web browser (i.e., no separate application download needed, because *Flow* is deployed *via* the WebXR platform).

**Figure 2:**
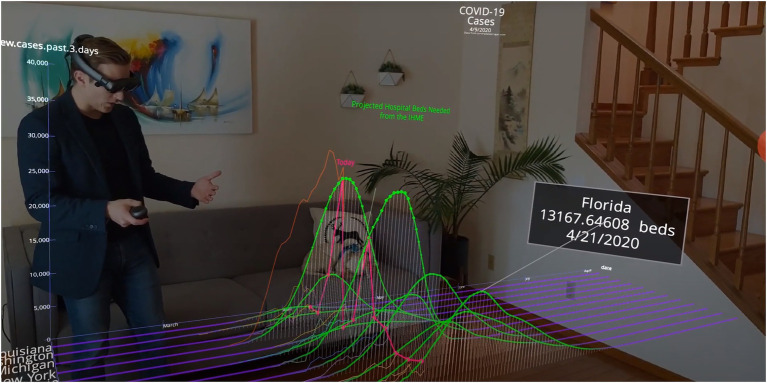
Example of a predictive simulation of augmented reality (AR) Covid-19 data for multiple states in the United States. The *X*-axis is time, the month of March is visible by the presenter’s leg; the *Y*-axis is number of new cases based on previous 3 days, and the *Z*-axis is actually a series of lines representing multiple individual states in the United States.

Over the course of the 11-month timeframe since we began publishing Covid-19 data simulations, many design decisions were made intending to manage the tension between balancing reach (i.e., number of views) and engagement (i.e., active time spent). Here, we chronicle some of that journey from the Covid-19 simulations introduction in late January, 2020 to the simulations current usage – as of early January 2021. Figure 2 showcases the story of how different states have different predicted peak hospitalization times. Although data dashboards provide ample details, *Flow’s* immersive and interactive data stories may be a better method for guiding non-experts to understanding the insights in the data. The “story” is about how different states have different predicted peak hospitalization dates. Here, we explore the first signs of the curve flattening in the United States, but history forewarned us with the 1918 Spanish Flu that the second and third waves caused more deaths than the first wave (not pictured here). The figure shows predictive models from the Institute for Health Metrics and Evaluation (IHME). Highlighted in this data story are predicted peak hospital bed utilizations. These differ depending on the individual state in the United States.

**Figure 3:**
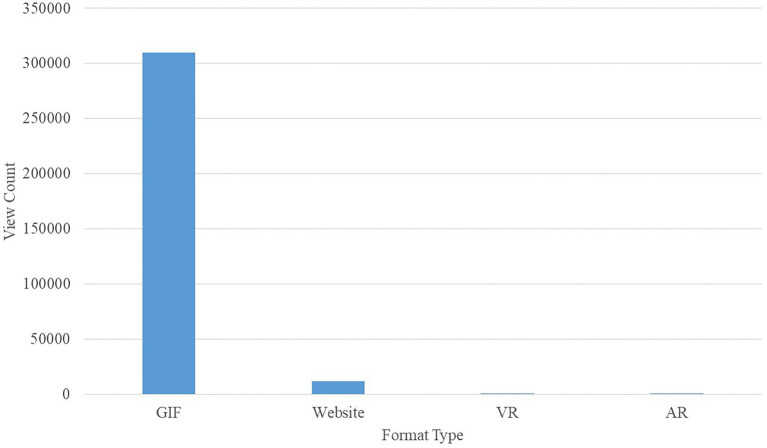
User reach in different formats for one Flow Immersive visualization.

In general, we saw that as the presentation format becomes more immersive (moving into augmented and virtual reality platforms), the reach (i.e., number of views) decreases and engagement (i.e., duration of the view) increases. Similarly, as the format becomes simpler and more accessible, the reach of the visualization increases and, yet, the engagement decreases. As an example, one of our early visualizations was made into an animated GIF and viewed over 310,000 times (Infinitemoment22, 2020), whereas the same visualization with user-controlled interactive capabilities on the website was viewed only 12,000 times. The numbers continue to drop as the platforms becomes less ubiquitous; around 300 people viewed it in VR headsets, and fewer than 20 people viewed it on AR headsets (the most commonly used AR platform was the *Magic Leap One*). Although the GIF duration was only 1 min, those in-headset spent an average of around 3 min exploring the content. The difference in terms of reach is illustrated in Figure 3.

## Usage in January: Beginning of the Covid-19 Pandemic

**Figure 4:**
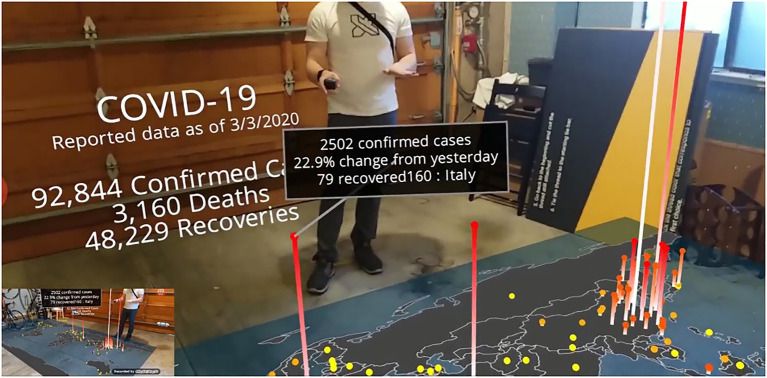
A snapshot from the AR presentation showing Covid-19 early March 2020 cases geospatially on an AR map. Height of spike corresponds to confirmed cases.

The GIF and interactive data visualization showing the spread of the disease over time appeared to be impactful. However, *via* viewer feedback, it became clear that the narrative is what really drives viewer engagement, rather than the visualization itself. The decision was made to rapidly record an AR session and to “walk the viewers through” the content with speech and the storyteller’s gestures. The vast majority of responses were positive and the number of views on *LinkedIn* quickly reached 2,000 in a non-advertised manner (DiBenigno, 2020a; Figure 4). Figure 4 shows that the relative impact of Covid-19 was mostly in China and Italy. Several viewers commented that the rate by which Italy was increasing was “shocking.” It would be more difficult to get these responses with a 2D heat map, especially for those who are colorblind.

Ten additional *Flows* were then created in this same spirit, averaging approximately 3 min in length. Videos were captured and shared *via* AR on a smartphone.

## The Amazing Reach Of *Tiktok*

**Figure 5:**
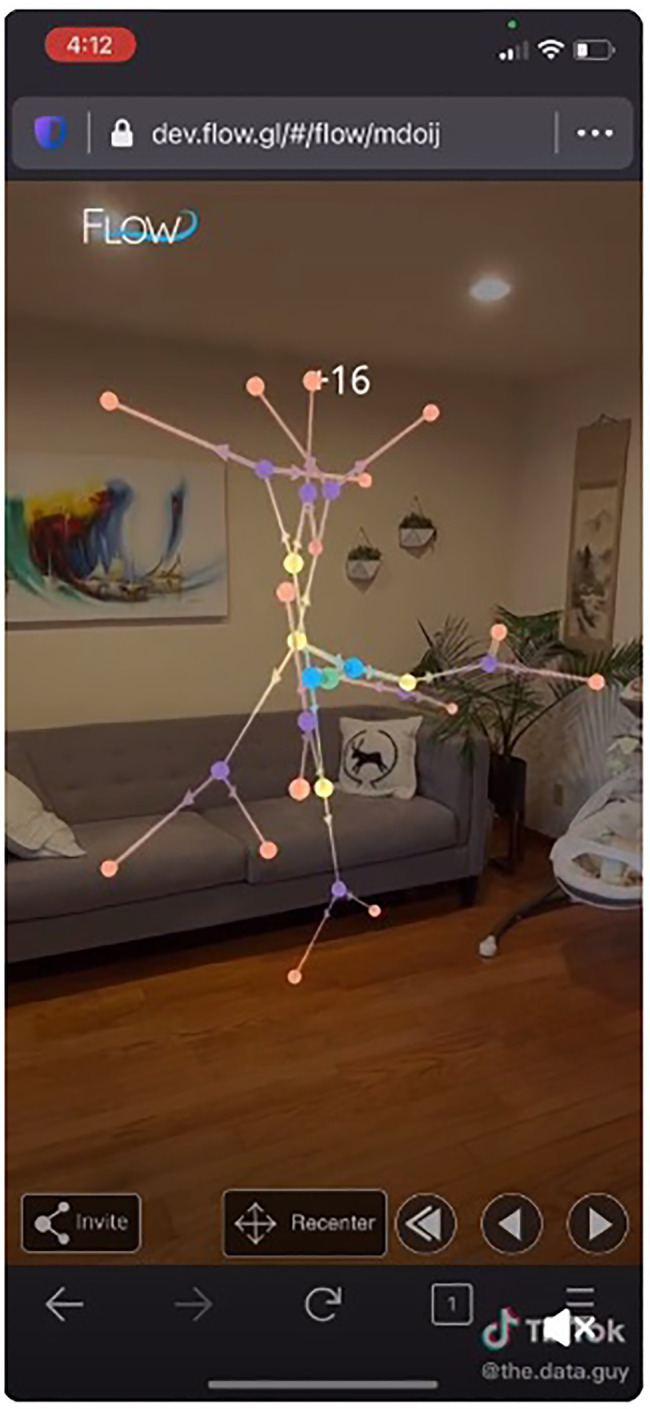
A snapshot from the *TikTok* animation illustrating how super-spreader events were driving Covid-19 infections. The two blue nodes represent the first two cases.

*TikTok* is a social media platform that is driven by algorithms that prioritize content virality over an established following. As a result, content has the possibility to reach a very large number of people without having a solidified following of viewers. This “viral”-focused platform enabled Flow Immersive to reach a much larger audience. One such example of how a Flow extended beyond its previous presentations is a *TikTok* video where disease propagation of Covid-19 is addressed (DiBenigno, 2020b). This *TikTok* video received over 2 million views over the span of 7 days (Figure 5). In this specific video, it is explained that in fact, 80–90% of Covid-19 infections is caused by 10–20% of people. This network graph allowed us to visualize the difference between a mathematical model for disease whereby each node spawns two additional nodes vs. a network graph where only 10% of the nodes was responsible for 80–90% of the spread, painting a much different picture.

**Figure 6:**
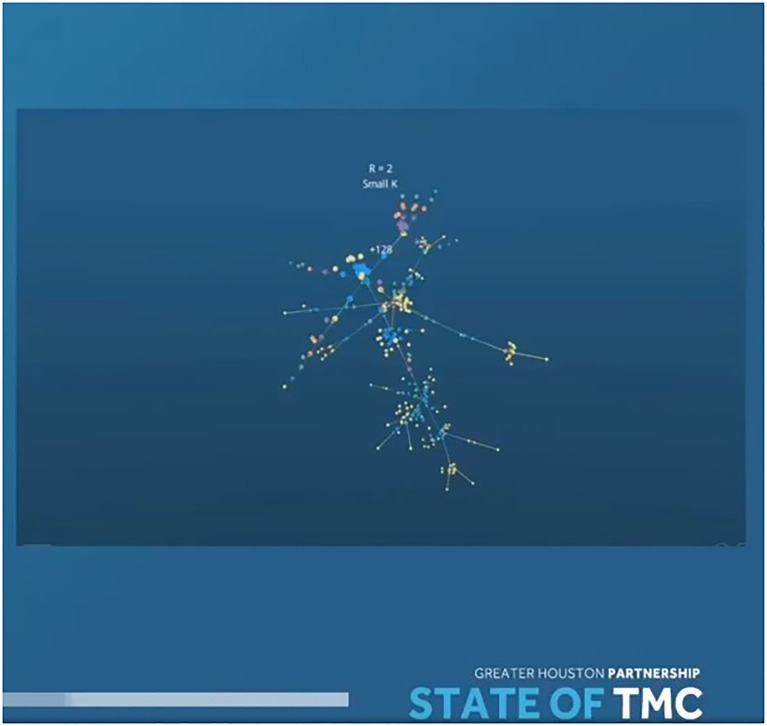
A YouTube snapshot from the Texas Medical Center’s “State of the TMC” event.

Soon thereafter, our disease transmission video was presented in a large flat-screen format by William McKeon, President and CEO Texas Medical Center. That *Flow* continues to live on the platform as an interactive visualization (Texas Medical Center, 2020; Figure 6). The Medical Center story was similar. The presenter walked viewers through how a virus with a certain R naught can propagate through a population. He chose this as one of the best illustrations of how disease spreads and why it is important to stop super spreader events.

## Flow Immersive Usage: End of 2020

With the exponential reach of our videos featuring AR *via TikTok*, we felt more empowered to explore content on XR devices. Instead of conceiving of *Flow* as one specific format, we designed it to be used as a funnel to draw people into more and more immersive experiences. Users claimed that our system was intuitive and helpful. Over the course of the year, *Flows* have been viewed over 9 million times.^3^ Below is an unsolicited, posted anecdote from a user in an AR headset (Lang, 2020):

*“… Using a spatial visualization like this and stepping through day by day supplements our intuitive perception and makes the difference obvious…”*

**Figure 7:**
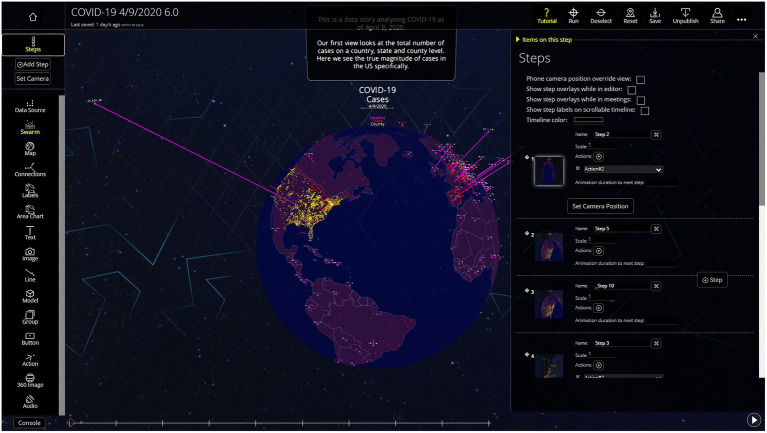
Flow Immersive Web-Based Editor. This is how all of the visualizations and interactive experiences are created.

The team at Flow Immersive is currently refining the web-based editor that allows anyone to build their own data story. There is growing interest, and an expanding waitlist, for the future Flow Immersive Editor. The Editor allows anyone without coding skills to enter data and begin creating a “story.” The Flow Immersive team is currently ramping up support for this feature. Figure 7 shows a screenshot of the Editor.

The largest takeaway from this endeavor is that there is a strong desire in the population (which includes a non-technical audience) to engage with, present, and really understand data. Epidemiologists often have the right information, but if it is not effectively communicated, it loses public health value. *Flow* is a pioneering method to increase engagement around 3D data visualizations and personalize presentations. We acknowledge that visualizing data in AR/VR has its own challenges, such as screen limitations and tailored interface design (Olshannikova et al., 2015), and we aim to overcome some of those in our future work.

## Qualitative User Feedback

To better understand *Flow’s* uses, a survey was sent to 40 people in March 2020. A total of 16 consented to evaluate the tool with the survey consisting of open-ended questions. One of the participants stated that they did not experience *Flow* with Covid-19 content and, therefore, was not included below. Of the 15, nine participants stated that they had specific Covid-19 questions they wanted answered within *Flow*. Among the ones that had prior Covid-19 questions in mind, the most common were: comparison by state (latest data and over time), geographical vaccine distribution, cities with high infection rates, comparison with other countries, and the results of hypothetical cases where stricter measures were taken. Table 1 highlights some of the results.

**Table 1:** Survey information of interest from 15 responders.

Type of experience	Longer form video	12
	Shorter form video	11
Type of platform	Phone	6
	Desktop	7
	VR	2
	AR	2
Specific Covid-19 related questions?	Yes	9
	No	6

When queried how *Flow* is different from other, more traditional and non-interactive visualizations, two clusters of answers emerged: (1) quality of interaction and (2) learning. Users found *Flow* to be a rich experience thanks to the storytelling aspects with personalized experiences. Some illustrative replies are listed below:

1. Quality of Interaction:
  “Made me watch it longer than I would have, more memorable.”
  “A *Flow* chart immerses you in the content!”
  “It was nice to see the graphs in such an immersive way.”
2. Learning:
  “Much richer learning experience.”
  “More digestible data can be one screen.”
  “It simplifies my understanding.”
  “Greater understanding, easier to remember.”
  “See data in a new way.”
  “Liked how the time aspect was displayed by real-time graph evolution.”

When asked how *Flow* helped them, participants’ reactions were uniformly positive. They found *Flow* to be visually pleasing, entertaining, and easy to use in general:

“Visually engaging”

“Great graphics”

“It’s entertaining more than 2d graphs”

“Visualizations were also clear and easy to read.”

Lastly, regarding the future of Flow Immersive, some responders asked to see its integration into other platforms, like multi-user AR. We are exploring how to get all users interactive in a multi-user space. They also asked for extrapolations and predictions, and the ability to direct attention to certain local data points:

“Integrations into enterprise data visualization platforms with tools to create and publish stories with the data.”

“Would be cool for two people in different locations to virtually occupy the same AR space.”

“If you speak about certain data points have those visually ‘flash’ to quickly help viewer find them.”

## Future Work

It has been anecdotally reported that some people hold the misconception that by simply putting data in a 3D space, major insights will instantly be grasped by the user. In fact, the affordances of 3D can lead to cognitive overload and confusion when the content is not well-scaffolded (Johnson-Glenberg, 2018). Nonetheless, thoughtful design that takes into account the special affordances of 3D space, should result in an increase in comprehension for spatially complex content. We posit that it is possible to dynamically show both the forest and the trees, i.e., the big picture, as well as the minute details.

The future for Flow Immersive is less about self-produced content to draw interest, and is more about empowering anyone to be able to tell their own data story with the editor. It is data democratization. With the recent wave of more affordable AR and VR devices, viewing 3D content is becoming more prevalent. A user going into a 3D environment to simply look at 2D content could be missing rich and vital information. VR headset adoption is slowly picking up. While the majority of users view *Flows* on flat screens, or in AR mode on mobile devices, with time, users will be able to create and then experience the data in a more immersive manner.

## Conclusion

This article introduces Flow Immersive – an interactive multi-user, multi-dimensional, multi-platform data storytelling application. During the Covid-19 pandemic, it has been used by millions to better understand disease transmission. Although not everyone had the opportunity to experience the simulations in emerging AR or VR, the tool still managed to capture wide audience attention *via* other formats. The tool shows promise in terms of engagement and understanding, especially when people are “immersed in data.” This is because as the format becomes more immersive, the engagement (time in the experience) also increases. The exponential increase in use afforded by the *TikTok* platform was unforeseen, yet exciting. The novel technology of *Flow* is being increasingly used by stakeholders and the wider public to understand, and to make decisions about Covid-19. These sorts of data visualizations hold promise for all types of data and may arguably result in increased data visualization literacy over time.

## Data Availability Statement

The original contributions presented in the study are included in the article/supplementary material, further inquiries can be directed to the corresponding authors.

## Ethics Statement

Written informed consent was obtained from the individual(s) for the publication of any potentially identifiable images or data included in this article.

## Author Contributions

MD: tool design and implementation and overall writing. MK: literature writing and reviewing. MJ-G: reviewing and supervising. All authors contributed to the article and approved the submitted version.

### Conflict of Interest

MD is the cofounder of the company Flow Immersive.The remaining authors declare that the research was conducted in the absence of any commercial or financial relationships that could be construed as a potential conflict of interest.

## Footnotes

^1^*Flow* also has focused on helping enterprises better communicate with data and has been used by organizations such as the UNDP, The World Bank, Deloitte, and BlackRock.

^2^https://a.Flow.gl/#/Flow/5zsc0ky

^3^Example situations we are aware of include institutions and news channels [e.g., internally at Institute for Health Metrics and Evaluation (IHME) and at Spectrum News 1 in North Carolina; Wu, 2020].
